# Long-Term Outcomes and Risk Factors for Treatment Failure After Internal Fixation of Femoral Neck Fractures: A 5-Year Follow-Up Study

**DOI:** 10.3390/jcm15218656

**Published:** 2026-11-06

**Authors:** Shuo Chen, Shangwei Ji, Haohao Xu, Renwei Cao, Yujiang Mao, Yejun Zha, Maoqi Gong, Xieyuan Jiang

**Affiliations:** 1 Peking University Fourth School of Clinical Medicine, Beijing 100083, China; 2 Department of Traumatology and Orthopedics, Beijing Jishuitan Hospital, Capital Medical University, Beijing 100035, China

**Keywords:** femoral neck fracture, internal fixation, complications, ONFH, treatment failure

## Abstract

**Objectives:** We sought to characterize long-term outcomes following internal fixation of femoral neck fractures (FNFs) in a consecutive cohort and to examine factors related to treatment failure. **Methods:** The study comprised 427 consecutive patients treated with internal fixation for FNFs at Beijing Jishuitan Hospital during 2017. Long-term outcomes were assessed through medical-record review, available routine imaging, and telephone follow-up during the fifth postoperative year. The primary long-term outcomes were osteonecrosis of the femoral head (ONFH) and major reoperation. Risk factor analysis was performed in 234 patients with radiographic follow-up of ≥6 months, in whom treatment failure was defined as a composite of ONFH, major reoperation, malunion, nonunion, and/or fixation failure. Associations between candidate factors and treatment failure were examined using univariable and multivariable logistic regression. **Results:** Long-term outcome status was successfully ascertained in 373 of 427 patients (87.4%), corresponding to a loss-to-follow-up rate of 12.6%. Overall, 98 patients (26.3%) developed ONFH and 57 (15.3%) underwent major reoperation, all of which were hip arthroplasties. Among the 234 patients with radiographic follow-up, 111 (47.4%) experienced treatment failure. In the multivariable analysis, high-energy injury (adjusted odds ratio [aOR] 2.90, 95% CI 1.06–7.92), displaced fracture (aOR 12.03, 95% CI 4.33–33.40), subcapital fracture location (aOR 2.14, 95% CI 1.04–4.41), and fair-to-poor reduction quality (aOR 3.95, 95% CI 1.88–8.31) were independently associated with treatment failure. In exploratory age-stratified analyses, fracture displacement and fair-to-poor reduction quality remained consistently associated with treatment failure in both age groups. **Conclusions:** This consecutive real-world cohort achieved long-term outcome ascertainment in 87.4% of the original population. Long-term complications after internal fixation of FNFs remained substantial. Fracture displacement and fair-to-poor reduction quality were the most consistent factors associated with treatment failure across the primary and exploratory age-stratified analyses, emphasizing the importance of careful treatment selection and adequate fracture reduction.

## 1. Introduction

Femoral neck fractures (FNFs) remain one of the most challenging injuries in orthopaedic trauma because treatment decisions must balance fracture healing, preservation of the femoral head, and restoration of function. Internal fixation remains an important treatment strategy, particularly for younger patients and selected older patients with nondisplaced fractures, in whom preservation of the native hip is preferred [1,2]. However, despite advances in fixation techniques and perioperative management, complications such as osteonecrosis of the femoral head (ONFH), nonunion, fixation failure, and subsequent reoperation remain substantial concerns after internal fixation [1,3,4,5].

Previous studies have investigated outcomes after internal fixation of FNFs. However, reported complication rates vary considerably because of differences in patient selection, fracture characteristics, follow-up duration, and outcome definitions. Many previous cohorts have focused on specific populations, such as young adults with high-energy injuries or elderly patients with nondisplaced fractures, which may limit the generalizability of their findings to routine clinical practice [1,5,6,7,8]. Furthermore, long-term outcome studies often rely on patients with available radiographic follow-up, potentially introducing selection bias because patients returning for prolonged imaging may not represent the entire treated population.

Large consecutive real-world cohorts with comprehensive long-term outcome assessment after internal fixation remain limited. Beijing Jishuitan Hospital, serving as the National Orthopaedic Medical Center, provides an opportunity to evaluate outcomes across a broad spectrum of patients treated in routine clinical practice. By combining long-term clinical follow-up with radiographic evaluation in a predefined subgroup, this study aimed to provide a more comprehensive assessment of treatment outcomes and associated risk factors after internal fixation of FNFs.

Therefore, this study had two objectives: (1) To evaluate long-term clinical outcomes, including ONFH and major reoperation, in a consecutive cohort of patients undergoing internal fixation for FNFs. (2) To identify risk factors associated with treatment failure using detailed radiographic follow-up data.

## 2. Method

### 2.1. Study Population

We retrospectively reviewed all patients who underwent internal fixation for FNFs at Beijing Jishuitan Hospital between 1 January and 31 December 2017.

Inclusion criteria included the following:

1. Patients with acute or subacute FNFs who underwent internal fixation at Beijing Jishuitan Hospital from 1 January 2017 to 31 December 2017;
2. Complete baseline clinical and radiographic data.

Exclusion criteria included the following:

1. Pathological fracture;
2. Combined with acetabular, peritrochanteric, or femoral head fractures or hip dislocations;
3. Skeletally immature patients;
4. Previous surgery on the contralateral hip.

### 2.2. Data Collection and Radiographic Evaluation

Demographic characteristics, clinical variables, and outcome information were extracted from inpatient and outpatient records in the institutional information system, with telephone contact used to supplement follow-up data. Medical factors associated with fracture healing and ONFH were identified [8,9,10], including chronic smoking, chronic alcohol abuse, steroid use, diabetes, end-stage renal disease, chronic bone metabolic disorders, and autoimmune diseases.

All available imaging—including plain radiographs, CT, MRI, and the corresponding radiology reports—was reviewed. Two orthopedic trauma surgeons independently evaluated preoperative (“injury”), intraoperative, and postoperative (including follow-up) radiographs. In case of disagreement, a third orthopedic trauma surgeon adjudicated.

Injury characteristics included injury mechanism (high- versus low-energy), initial displacement (modified Garden classification [11,12]), anatomical classification and modified Pauwels classification [13]. Surgical factors included time to surgery, reduction method, reduction quality, and implant type. Fracture comminution was assessed on preoperative radiographs and CT images.

Reduction quality was graded according to previously published criteria [14]. A reduction was considered good when residual displacement was ≤2 mm, posterior angulation on the lateral view was ≤10°, and the Garden angle on the anteroposterior view was between 160° and 175°. Reductions with ≤5 mm of displacement and ≤20° of posterior angulation while retaining a Garden angle of 160–175° were categorized as fair. Reduction was classified as poor when displacement exceeded 5 mm, posterior angulation exceeded 20°, or the Garden angle fell outside the 160–175° range.

### 2.3. Outcome Assessment

Clinical outcomes were evaluated in all eligible patients through review of medical records, available radiographic data obtained during routine clinical follow-up, and telephone follow-up performed during the fifth postoperative year. The primary clinical outcomes were ONFH and major reoperation.

1. ONFH status was ascertained using all available clinical information, including radiographs, CT or MRI examinations, radiology reports, medical records, and telephone follow-up. When adequate imaging was available, ONFH was classified according to the ARCO classification system [15].
2. Major reoperation was defined as early revision of fixation, proximal femoral osteotomy, or hip arthroplasty.

For risk factor analysis, only patients with radiographic follow-up of at least 6 months were included. Patients without adequate radiographic follow-up were excluded from risk factor analysis. In this subgroup, treatment failure served as the outcome for risk factor analysis and was defined as a composite outcome including ONFH, major reoperation, malunion, nonunion and/or fixation failure.

**Figure 1:**
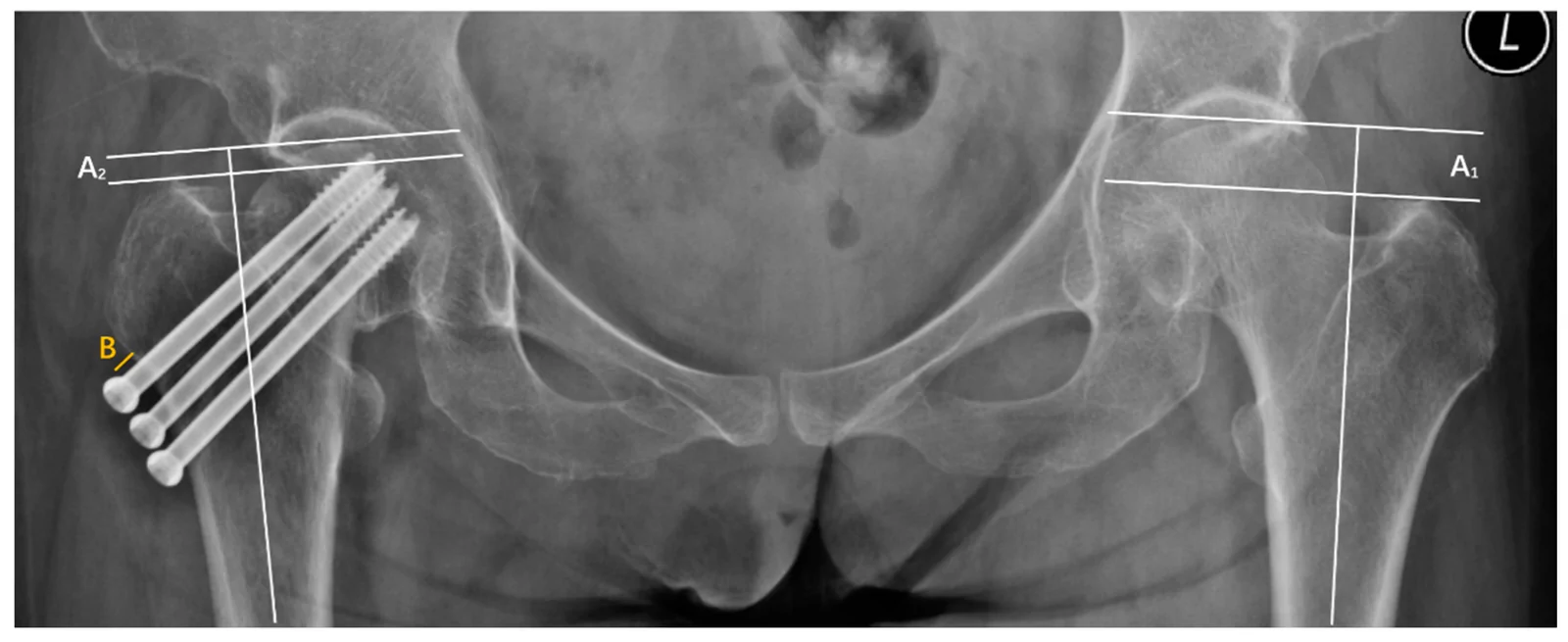
Radiographic measurement of femoral-neck shortening [8]. Method A compares the magnification-adjusted vertical relationship between the femoral head and greater trochanter on the contralateral (A_1_) and injured (A_2_) sides. Method B quantifies shortening from lateral implant prominence (B). The larger absolute measurement obtained with the two approaches was used for analysis.

3. Malunion was defined as a shortening of the vertical or femoral neck of ≥10 mm. The maximum absolute value for “shortening” was defined using 2 methods, as shown in Figure 1 [8,16,17]. Since there is no recognized benchmark for clinically femoral neck shortening and the desire to maintain a native hip varied among patients in this cohort, we also calculated the incidence of shortening with a threshold of ≥15 mm [8].
4. Nonunion and/or fixation failure was defined as lack of union >6 months and/or loss of mechanical integrity of the implant [18,19].

### 2.4. Statistical Analysis

Analyses were conducted using IBM SPSS Statistics version 27.0 (IBM Corp, Armonk, NY, USA).

Continuous variables are reported as mean ± standard deviation, and categorical variables as number and percentage. Between-group comparisons used Student’s *t* test for continuous measures and Pearson’s chi-square or Fisher’s exact test for categorical measures, as appropriate. All tests were two-sided, with *p* < 0.05 indicating statistical significance.

Univariable and multivariable binary logistic regression analyses were performed to identify independent risk factors for treatment failure. Covariates for the multivariable logistic regression were specified a priori on the basis of clinical relevance and previous evidence rather than selected according to univariable statistical significance alone.

## 3. Results

A total of 427 patients were included in the study. Of these, 418 (97.9%) were treated with cannulated screws, five (1.2%) with intramedullary nails, and four (0.9%) with dynamic hip screws. At the 5-year follow-up, 54 patients were lost to follow-up, resulting in a loss-to-follow-up rate of 12.6%. Thus, 373 patients were available for final analysis (Table 1), including 184 elderly patients (49.3%), 241 female patients (64.6%), and 192 displaced fractures (51.5%).

Overall, 98 patients (26.3%) developed ONFH. ONFH was confirmed from available imaging in 79 patients and was reported during telephone follow-up in 19 patients. There were 57 cases of major reoperation related to complications, with an incidence rate of 15.3%, all of which were hip arthroplasties. The reasons for secondary surgeries included 1 case of fixation failure, 4 cases of nonunion (including 3 with concomitant ONFH), 50 cases of ONFH, 1 case of symptomatic malunion, and 1 case with an unknown indication identified during telephone follow-up. The average time to major reoperation was 32.6 months postoperatively. In addition, 24 patients had internal fixation removed after surgery.

We divided the patients into groups based on an age threshold of 60 years and evaluated their clinical characteristics and treatment outcomes (Table 2). A cutoff of 60 years was selected because internal fixation remains the preferred treatment strategy in many patients younger than 60 years. Among the 189 young patients, 60 (31.7%) developed ONFH, and 28 (14.8%) subsequently underwent hip arthroplasty. Among the 184 elderly patients, 38 (20.7%) developed ONFH and 29 (15.8%) underwent hip arthroplasty.

Considering that radiographic evaluation is an important component of outcome assessment, we screened patients with radiographic follow-up of at least 6 months for more detailed evaluation of clinical outcomes and risk factor analysis. This subgroup consisted of 234 patients with a mean radiographic follow-up of 23.6 ± 15.1 months. The median was 20.5 months (interquartile range 12.0–34.0 months; range 6–63 months). Similarly, we divided these 234 patients into young and elderly groups and found significant differences in baseline characteristics, including injury mechanism, sex, osteoporosis, fracture displacement, and fracture comminution (Table 3). However, no significant differences in treatment outcomes were observed between the two age groups (Table 4). Among these 234 patients, treatment failures included malunion in 39 patients (16.7%), ONFH in 87 patients (37.2%), and nonunion and/or fixation failure in 11 patients (4.7%). A total of 50 patients (21.4%) underwent major reoperation. Fifty-eight patients (24.8%) experienced two or more components of the composite endpoint. Interobserver agreement was 91.0% for fracture displacement (κ = 0.816; 95% CI 0.742–0.891) and 86.3% for reduction quality (linearly weighted = 0.746; 95% CI 0.662–0.830).

Table 5 presents the univariable and multivariable logistic regression analyses of treatment failure in the radiographic follow-up cohort. In univariable analyses, higher BMI (OR 1.09, 95% CI 1.00–1.18, *p* = 0.040), displaced fracture (OR 13.36, 95% CI 6.82–26.19, *p* < 0.001), increasing Pauwels angle (OR 1.05 per degree, 95% CI 1.02–1.08, *p* < 0.001), fracture comminution (OR 6.37, 95% CI 3.57–11.37, *p* < 0.001), and fair-to-poor reduction quality (OR 5.91, 95% CI 3.14–11.13, *p* < 0.001) were associated with treatment failure.

In the multivariable model, high-energy injury (adjusted OR [aOR] 2.90, 95% CI 1.06–7.92, *p* = 0.038), displaced fracture (aOR 12.03, 95% CI 4.33–33.40, *p* < 0.001), subcapital fracture (aOR 2.14, 95% CI 1.04–4.41, *p* = 0.039), and fair-to-poor reduction quality (aOR 3.95, 95% CI 1.88–8.31, *p* < 0.001) were independently associated with treatment failure. Age showed a borderline association with treatment failure (aOR 1.03 per year, 95% CI 1.00–1.05, *p* = 0.054), whereas sex, BMI, Pauwels angle, and fracture comminution were not independently associated with failure after adjustment.

In exploratory age-stratified multivariable analyses, displaced fracture and fair-to-poor reduction quality were consistently associated with treatment failure in both age groups (Table 6). Among patients aged <60 years, displaced fracture (aOR 21.48, 95% CI 5.86–78.71, *p* < 0.001), fair-to-poor reduction quality (aOR 2.88, 95% CI 1.05–7.93, *p* = 0.041), and increasing age (aOR 1.04 per year, 95% CI 1.00–1.07, *p* = 0.028) were associated with treatment failure. Among patients aged ≥60 years, displaced fracture (aOR 14.21, 95% CI 3.96–51.01, *p* < 0.001) and fair-to-poor reduction quality (aOR 5.82, 95% CI 1.99–16.99, *p* = 0.001) remained associated with treatment failure. Subcapital fracture showed a borderline association in the older subgroup (aOR 2.99, 95% CI 0.95–9.35, *p* = 0.060).

In exploratory outcome-specific analyses, displaced fractures were associated with both ONFH and major reoperation. Fair-to-poor reduction quality was associated with ONFH but did not reach statistical significance for major reoperation. Female sex was associated with ONFH (aOR 3.00; 95% CI 1.36–6.62) and major reoperation (aOR 4.05; 95% CI 1.62–10.16). Increasing age was also associated with major reoperation (aOR 1.07 per year; 95% CI 1.02–1.11).

## 4. Discussion

Femoral neck fractures treated with internal fixation remain associated with substantial long-term complications. In the present consecutive cohort, 373 of 427 patients (87.4%) were available for 5-year outcome assessment. ONFH occurred in 26.3% of patients, and 15.3% underwent major reoperation. All major reoperations were hip arthroplasties, with ONFH accounting for most of these procedures. These findings indicate that clinically important complications remain common after internal fixation despite contemporary treatment strategies.

Previous studies have reported considerable variation in outcomes after internal fixation of FNFs. Reported rates of osteonecrosis, nonunion, fixation failure, and reoperation differ substantially according to patient age, fracture displacement, treatment strategy, duration of follow-up, and outcome definitions [1,6,7,8,20]. Slobogean et al. [1] reported a pooled reoperation rate of 18.0% in young adults, whereas Collinge et al. [8] reported a major reconstructive procedure rate of 32% in a multicenter cohort of young patients. In elderly patients with nondisplaced or minimally displaced fractures, a meta-analysis by Overmann et al. [6] reported a pooled reoperation risk of 14.1%. Direct comparison among these studies is difficult because of substantial heterogeneity in patient selection and outcome definitions.

An important strength of the present study is the high level of long-term outcome ascertainment in a consecutive real-world cohort from Beijing Jishuitan Hospital (National Orthopaedic Medical Center). All eligible consecutive patients who underwent internal fixation for FNFs during a single calendar year were included, and long-term outcome status was successfully ascertained in 373 of 427 patients (87.4%), corresponding to a loss-to-follow-up rate of only 12.6%. Unlike many previous studies focusing on selected populations, such as young adults or older patients with nondisplaced fractures, the present study included all eligible consecutive patients treated with internal fixation during the study period, regardless of age or fracture displacement. Moreover, long-term outcome ascertainment was not dependent on patients returning for prolonged radiographic follow-up. This distinction is clinically relevant because the subgroup with ≥6 months of radiographic follow-up showed higher observed rates of ONFH and major reoperation than the overall cohort, illustrating the potential selection bias introduced when long-term outcomes are estimated only from patients with extended imaging follow-up. The combination of long-term clinical follow-up and a separate radiographic subgroup allowed us to describe overall long-term outcomes while also examining radiographic complications and associated factors in greater detail.

Younger and older patients differed substantially in their injury characteristics. Younger patients were more likely to sustain high-energy injuries and displaced or comminuted fractures, whereas older patients were more frequently female and had osteoporosis. Nevertheless, no statistically significant differences in treatment failure, ONFH, nonunion or fixation failure, malunion, or major reoperation were observed between the two age groups in the radiographic follow-up cohort. The absence of statistically significant differences does not establish equivalent outcomes between the age groups. In the overall 5-year cohort, ONFH was numerically more frequent in patients aged <60 years, whereas the rates of major reoperation were similar between the two age groups. These findings emphasize that substantial complications occur in both younger and older patients selected for internal fixation.

In the radiographic follow-up cohort, univariable analyses showed associations of higher BMI, fracture displacement, increasing Pauwels angle, fracture comminution, and fair-to-poor reduction quality with treatment failure. After multivariable adjustment, high-energy injury, displaced fracture, subcapital fracture location, and fair-to-poor reduction quality remained independently associated with treatment failure. In contrast, BMI, Pauwels angle, and fracture comminution were no longer independently associated with failure after adjustment. Age showed a borderline association in the primary model.

Among these findings, fracture displacement and reduction quality were the most consistent factors across the primary and exploratory age-stratified analyses. Displaced fractures were strongly associated with treatment failure in both patients aged <60 years and those aged ≥60 years. This finding is consistent with previous studies showing that initial fracture displacement is an important determinant of failure after internal fixation [8,21,22,23,24,25]. Greater displacement may reflect more severe disruption of the vascular supply to the femoral head as well as greater mechanical instability, both of which may adversely affect fracture healing and femoral-head viability.

Reduction quality was also independently associated with treatment failure in the overall cohort and in both age groups. Unlike many patient- and injury-related characteristics, reduction quality represents a potentially modifiable factor. Previous studies have similarly emphasized the importance of achieving adequate reduction before definitive fixation [8,14,21,22,23,24,25]. Restoration of fracture alignment and stable fixation may improve the mechanical environment for healing and may facilitate preservation or restoration of the femoral-head blood supply. Taken together, these findings reinforce the importance of obtaining the best possible reduction, particularly in displaced fractures.

High-energy injury and subcapital fracture location were independently associated with treatment failure in the multivariable model. High-energy injury was not included in the exploratory age-stratified multivariable models, whereas the association with subcapital fracture location was less consistent across age groups. Subcapital fracture was not associated with failure in patients aged <60 years and showed only a borderline association in patients aged ≥60 years. These findings should therefore be interpreted cautiously and require confirmation in larger cohorts. Similarly, the age-stratified analyses were exploratory and produced relatively wide confidence intervals, limiting the precision of subgroup-specific effect estimates.

The composite endpoint captures adverse outcomes with differing clinical implications. The association between age and major reoperation may partly reflect treatment preferences. The sex estimates changed after adjustment for fracture characteristics and should be interpreted cautiously.

Implant type could not be meaningfully evaluated in the present study because the great majority of fractures were treated with multiple cannulated screws, with very few patients receiving alternative fixation devices. The results should therefore primarily be interpreted in the context of cannulated-screw fixation, and whether newer fixed-angle devices provide different long-term outcomes requires further investigation. These findings should also be considered alongside evidence comparing internal fixation with arthroplasty. A network meta-analysis favored arthroplasty over cannulated screw fixation for reoperation risk and functional outcomes [26].

Several limitations should also be acknowledged. First, the retrospective design introduces the possibility of treatment-selection bias, because patients selected for internal fixation differ from those treated with arthroplasty. Second, ONFH ascertainment in patients without long-term radiographic follow-up depended partly on patient-reported information and available medical records and may therefore underestimate the true incidence. Radiographic follow-up duration varied among patients. Some complications requiring imaging assessment may have been missed, including malunion. Third, the radiographic subgroup was subject to selection bias and was smaller than the overall cohort. The heterogeneity of the composite endpoint also limits interpretation and the exploratory outcome-specific estimates remain subject to residual confounding. Finally, this was a single-center study in which most fractures were treated with cannulated screws, which may limit generalizability to other treatment settings and fixation strategies.

In conclusion, this study provides 5-year real-world outcome data from a consecutive cohort treated at a National Orthopaedic Medical Center, with long-term outcome status successfully ascertained in 87.4% of the original cohort. Long-term complications after internal fixation of FNFs remained substantial in this consecutive cohort. Fracture displacement and fair-to-poor reduction quality were the most consistent factors associated with treatment failure across the primary and exploratory age-stratified analyses. High-energy injury and subcapital fracture location were additionally associated with treatment failure in the primary adjusted model. These findings support careful assessment of fracture characteristics when selecting treatment and emphasize the importance of achieving adequate reduction and maintaining long-term follow-up after internal fixation.

## Figures and Tables

**Table 1:** Description of the Overall Cohort (*n* = 373).

Variable	*n* (%)
Elderly age group (≥60 y.o.)	184 (49.3%)
Female	241 (64.6%)
Displaced fractures	192 (51.5%)
ONFH	98 (26.3%)
Major reoperation	57 (15.3%)

**Table 2:** Baseline data and clinical outcomes among two age groups.

	Younger Age Group (<60 y.o.)	Elderly Age Group (≥60 y.o.)
	(*n* = 189)	(*n* = 184)
high-energy injury *	47 (24.9%)	11 (6%)
Female	88 (46.6%)	153 (83.2%)
Osteoporosis	10 (5.3%)	83 (45.1%)
Displaced fractures	135 (71.4%)	57 (31%)
Fracture comminution	97 (51.3%)	31 (16.8%)
ONFH	60 (31.7%)	38 (20.7%)
Major reoperation	28 (14.8%)	29 (15.8%)

* High-energy injury including fall from a height, ice skating, traffic accident, motorcycle accident and electric vehicle accident.

**Table 3:** Patient and injury characteristics in 234 patients with postoperative imaging follow-up of ≥6 months.

Variable	Entire Cohort	Younger Age Group (<60 y.o.)	Older Age Group (≥60 y.o.)	*p*
Number of patients (*n*)	234	136	98	NA
High-energy injury	40 (17.1%)	33 (24.3%)	7 (7.1%)	<0.001
Time to surgery (d)	4.9 ± 4.8	4.7 ± 5.1	5.0 ± 4.3	0.64
Female gender	143 (61.1%)	61 (44.9%)	82 (83.7%)	<0.001
Mean age (y)	55.5 ± 16.2	45.8 ± 12.7	69.1 ± 9.5	NA
Mean body mass index (BMI)	23.1 ± 3.3	23.2 ± 3.5	22.9 ± 3.0	0.543
Osteoporosis	50 (21.4%)	9 (6.6%)	41 (41.8%)	<0.001
Metabolic bone conditions, total	99 (42.3%)	60 (44.1%)	39 (39.8%)	0.509
Smoking	57 (24.4%)	44 (32.4%)	13 (13.3%)	<0.001
Alcohol misuse	33 (14.1%)	27 (19.9%)	6 (6.1%)	0.003
Diabetes mellitus	40 (17.1%)	14 (10.3%)	26 (26.5%)	0.001
Chronic steroid use	1 (0.4%)	1 (0.7%)	0 (0%)	
End-stage renal disease	9 (3.8%)	0 (0%)	9 (9.2%)	
Autoimmune diseases	9 (3.8%)	2 (1.5%)	7 (7.1%)	
Displaced fractures	139 (59.4%)	96 (70.6%)	43 (43.9%)	<0.001
Subcapital fracture	83 (35.5%)	49 (36%)	34 (34.7%)	0.833
Mean Pauwels angle (degrees)	46.9 ± 11.7	48.1 ± 11.4	45.2 ± 12.0	0.06
Pauwels classification				0.257
Type I	12 (5.1%)	6 (4.4%)	6 (6.1%)	
Type II	107 (45.7%)	57 (41.9%)	50 (51%)	
Type III	115 (49.1%)	73 (53.7%)	42 (42.9%)	
Fracture comminution	96 (41%)	71 (52.2%)	25 (25.5%)	<0.001
Associated femoral shaft fracture	3 (1.3%)	3 (2.2%)	0 (0%)	0.267

**Table 4:** Treatment characteristics and clinical outcomes in 234 patients with postoperative imaging follow-up of ≥6 months.

Variable	Entire Cohort	Younger Age Group (<60 y.o.)	Older Age Group (≥60 y.o.)	*p*
Number of patients (*n*)	234	136	98	NA
Reduction method				1
Closed	220 (94%)	128 (94.1%)	92 (93.9%)	
Open	14 (6%)	8 (5.9%)	6 (6.1%)	
Reduction quality				0.068
Good	163 (69.7%)	99 (72.8%)	64 (65.3%)	
Fair	57 (24.4%)	33 (24.3%)	24 (24.5%)	
Poor	14 (6%)	4 (2.9%)	10 (10.2%)	
Treatment failure *	111 (47.4%)	70 (51.5%)	41 (41.8%)	0.145
Treatment failure (malunion ≥ 15 mm)	98 (41.9%)	59 (43.4%)	39 (39.8%)	0.583
Treatment failure (excluding malunion)	92 (39.3%)	55 (40.4%)	37 (37.8%)	0.678
Nonunion and/or fixation failure	11 (4.7%)	8 (5.9%)	3 (3.1%)	0.367
Malunion (shortening ≥ 10 mm)	39 (16.7%)	27 (19.9%)	12 (12.2%)	0.123
ONFH	87 (37.2%)	53 (39%)	34 (34.7%)	0.504
Major reoperation	50 (21.4%)	24 (17.6%)	26 (26.5%)	0.102

* Treatment failure was defined as a composite outcome including ONFH, major reoperation, malunion with femoral neck shortening ≥10 mm, and nonunion and/or fixation failure.

**Table 5:** Univariable and multivariable logistic regression analyses of factors associated with treatment failure in 234 patients with postoperative imaging follow-up of ≥6 months.

Variable	Patients Without Failure (*n* = 123)	Patients With Failure (*n* = 111)	Unadjusted OR (95% CI)	*p* Value	Adjusted OR * (95% CI)	*p* Value
High-energy injury	16 (13.0%)	24 (21.6%)	1.84 (0.92–3.69)	0.083	2.90 (1.06–7.92)	0.038
Time to surgery, per day	5.1 ± 5.9	4.6 ± 3.1	0.98 (0.92–1.03)	0.393	—	—
Female sex	80 (65.0%)	63 (56.8%)	0.71 (0.42–1.20)	0.195	1.99 (0.86–4.59)	0.108
Age, per year	55.5 ± 18.2	55.6 ± 13.8	1.00 (0.98–1.02)	0.97	1.03 (1.00–1.05)	0.054
BMI, per kg/m^2^	22.7 ± 3.1	23.6 ± 3.4	1.09 (1.00–1.18)	0.04	1.04 (0.94–1.16)	0.451
Osteoporosis	31 (25.2%)	19 (17.1%)	0.61 (0.32–1.16)	0.134	—	—
Metabolic bone conditions	46 (37.4%)	53 (47.7%)	1.53 (0.91–2.58)	0.11	—	—
Displaced fracture	42 (34.1%)	97 (87.4%)	13.36 (6.82–26.19)	<0.001	12.03 (4.33–33.40)	<0.001
Subcapital fracture	39 (31.7%)	44 (39.6%)	1.41 (0.83–2.42)	0.206	2.14 (1.04–4.41)	0.039
Pauwels angle, per degree	44.0 ± 11.6	50.1 ± 11.0	1.05 (1.02–1.08)	<0.001	1.02 (0.98–1.05)	0.343
Fracture comminution	26 (21.1%)	70 (63.1%)	6.37 (3.57–11.37)	<0.001	1.79 (0.76–4.25)	0.185
Open reduction	4 (3.3%)	10 (9.0%)	2.95 (0.90–9.68)	0.075	—	—
Fair-to-poor reduction quality	17 (13.8%)	54 (48.6%)	5.91 (3.14–11.13)	<0.001	3.95 (1.88–8.31)	<0.001

* The multivariable model included age, sex, BMI, injury energy, fracture displacement, subcapital fracture location, Pauwels angle, fracture comminution, and reduction quality. Reference categories were low-energy injury, male sex, nondisplaced fracture, non-subcapital fracture, absence of fracture comminution, and good reduction quality. — indicates not included in the multivariable model. OR, odds ratio. CI, confidence interval.

**Table 6:** Age-stratified multivariable logistic regression analyses of factors associated with treatment failure.

Variable	<60 Years (*n* = 136; 70 Failures), Adjusted OR (95% CI) *	*p* Value	≥60 Years (*n* = 98; 41 Failures), Adjusted OR (95% CI) *	*p* Value
Age, per year	1.04 (1.00–1.07)	0.028	1.04 (0.97–1.11)	0.241
Displaced fracture	21.48 (5.86–78.71)	<0.001	14.21 (3.96–51.01)	<0.001
Subcapital fracture	1.46 (0.60–3.57)	0.405	2.99 (0.95–9.35)	0.06
Fair-to-poor reduction quality	2.88 (1.05–7.93)	0.041	5.82 (1.99–16.99)	0.001

* Multivariable models were fitted separately for patients aged <60 years and ≥60 years using the same four covariates: age, fracture displacement, subcapital fracture location, and reduction quality. These age-stratified analyses were exploratory secondary analyses. Complete age-stratified univariable regression results are provided in Supplementary Tables S1 and S2.

## Data Availability

The datasets analyzed for this study are available from the corresponding author upon reasonable request.

## Supplementary Materials

The following supporting information can be downloaded at https://www.mdpi.com/article/10.3390/jcm15218656/s1, Table S1: Univariable logistic regression analyses of factors associated with treatment failure in patients aged <60 years; Table S2: Univariable logistic regression analyses of factors associated with treatment failure in patients aged ≥60 years.
