# Supplementary material for: Long-Term Outcomes and Risk Factors for Treatment Failure After Internal Fixation of Femoral Neck Fractures: A 5-Year Follow-Up Study
Source: J Clin Med. 2026 Nov 6;15(21):8656. doi: 10.3390/jcm15218656 (PMC13763804; doi:10.3390/jcm15218656)
Supplement: Supplementary file 1 [file jcm-15-08656-s001.zip › jcm-4619566-supplementary.pdf]

Supplementary Table S1. Univariable logistic regression analyses of factors associated with treatment failure in patients aged <60 years.

Variable	Patients Without Failure (n=66)	Patients With Failure (n=70)	Unadjusted OR (95% CI)	P value
High-energy injury	14 (21.2%)	19 (27.1%)	1.38 (0.63–3.05)	0.421
Time to surgery, d	5.1 ± 6.9	4.4 ± 2.4	0.97 (0.91–1.04)	0.418
Female sex	33 (50.0%)	28 (40.0%)	0.67 (0.34–1.32)	0.242
Age, y	42.9 ± 13.9	48.4 ± 10.9	1.04 (1.01–1.07)	0.013
BMI, kg/m²	22.7 ± 3.4	23.7 ± 3.6	1.09 (0.98–1.20)	0.102
Osteoporosis	4 (6.1%)	5 (7.1%)	1.19 (0.31–4.65)	0.8
Metabolic bone conditions	22 (33.3%)	38 (54.3%)	2.37 (1.19–4.76)	0.015
Displaced fracture	29 (43.9%)	67 (95.7%)	28.49 (8.13–99.92)	<0.001
Subcapital fracture	21 (31.8%)	28 (40.0%)	1.43 (0.71–2.89)	0.321
Pauwels angle, degrees	44.9 ± 11.0	51.1 ± 11.0	1.05 (1.02–1.09)	0.002
Fracture comminution	20 (30.3%)	51 (72.9%)	6.17 (2.93–12.99)	<0.001
Open reduction	4 (6.1%)	4 (5.7%)	0.94 (0.23–3.92)	0.932
Fair-to-poor reduction quality	7 (10.6%)	30 (42.9%)	6.32 (2.53–15.79)	<0.001

Supplementary Table S2. Univariable logistic regression analyses of factors associated with treatment failure in patients aged ≥60 years.				
Variable	Patients Without Failure (n=57)	Patients With Failure (n=41)	Unadjusted OR (95% CI)	P value
High-energy injury	2 (3.5%)	5 (12.2%)	3.82 (0.70–20.76)	0.121
Time to surgery, d	5.1 ± 4.5	4.9 ± 4.0	0.99 (0.90–1.09)	0.8
Female sex	47 (82.5%)	35 (85.4%)	1.24 (0.41–3.74)	0.701
Age, y	70.0 ± 9.9	67.7 ± 8.8	0.97 (0.93–1.02)	0.24
BMI, kg/m²	22.6 ± 2.8	23.4 ± 3.2	1.09 (0.94–1.25)	0.244
Osteoporosis	27 (47.4%)	14 (34.1%)	0.58 (0.25–1.32)	0.192
Metabolic bone conditions	24 (42.1%)	15 (36.6%)	0.79 (0.35–1.81)	0.582
Displaced fracture	13 (22.8%)	30 (73.2%)	9.23 (3.65–23.33)	<0.001
Subcapital fracture	18 (31.6%)	16 (39.0%)	1.39 (0.60–3.21)	0.446
Pauwels angle, degrees	43.0 ± 12.3	48.3 ± 10.9	1.04 (1.00–1.08)	0.034
Fracture comminution	6 (10.5%)	19 (46.3%)	7.34 (2.58–20.88)	<0.001
Open reduction	0 (0.0%)	6 (14.6%)	Not estimable†	—
Fair-to-poor reduction quality	10 (17.5%)	24 (58.5%)	6.64 (2.64–16.70)	<0.001
